# C-reactive Protein Versus Procalcitonin in the Early Diagnosis of Neonatal Sepsis: A Systematic Review

**DOI:** 10.7759/cureus.90353

**Published:** 2025-08-17

**Authors:** Sri Vidya Sundara, Xinyu Lu, Hamide Busmail, Sasika Weerakoon, Sravanthi Avula, Bethel T Mandefro, Lubna Mohammed

**Affiliations:** 1 Pediatrics, California Institute of Behavioral Neurosciences & Psychology, Fairfield, USA; 2 Otolaryngology, Shanghai Pudong Hospital, Shanghai, CHN; 3 Internal Medicine, California Institute of Behavioral Neurosciences & Psychology, Fairfield, USA; 4 Pediatrics, NRI (Non-resident Indian) Medical College, Guntur, IND; 5 Internal Medicine, Dr Vizarath Rasool Khan (VRK) Women's Medical College, Hyderabad, IND

**Keywords:** antibiotic stewardship in nicu, antibiotic use in newborn, c-reactive protein (crp), early onset neonatal sepsis (eons), late onset neonatal sepsis (lons), neonatal sepsis, procalcitonin (pct), sepsis biomarkers, sepsis biomarker sensitivity, sepsis biomarker specificity

## Abstract

Neonatal sepsis is a systemic response to a bacterial infection within the first 28 days of life. Diagnosis remains a challenge due to the subtle and nonspecific signs, often resulting in the use of empirical antibiotics and their associated risks. This systematic review aims to compare the diagnostic accuracy and clinical utility of C-reactive protein (CRP) and procalcitonin (PCT) in the early diagnosis of neonatal sepsis. The review was conducted using our search strategy across PubMed/Medline, PubMed Central, Europe PMC, ScienceDirect, and Google Scholar. Eligible studies included free full-text systematic reviews, narrative reviews, diagnostic accuracy studies, and observational studies published in English between 2020 and 2025. Studies published in foreign languages, based on non-human studies, as well as grey literature that lacked free full-text articles, were excluded from the analysis. The risk of bias was assessed using appropriate quality assessment tools. Of the 16 initially eligible studies, 12 met the inclusion criteria after quality assessment; these consisted of eight narrative reviews, three diagnostic accuracy studies, and one observational study. Our systematic review found that although CRP is widely utilized due to its low cost and simplicity, its limited early sensitivity and susceptibility to non-infectious factors reduce its utility in diagnosing neonatal sepsis. In contrast, PCT demonstrated an earlier rise, correlates with the severity of the disease, has higher sensitivity (reported as 97.6 and 97.7% in two studies), and can differentiate between bacterial, viral, and fungal infections. However, PCT displays a physiological rise, can be influenced by non-infectious factors, and is costlier. In both cases, serial measurements and the use of adjusted nomograms enhance their use. In conclusion, PCT demonstrates superior diagnostic potential and utility in guiding antibiotic therapy compared to CRP for early diagnosis of neonatal sepsis, especially when used in combination with other biomarkers. As part of our review, we noted the absence of procalcitonin in current international neonatal sepsis guidelines, and we have proposed a flowchart to guide its potential integration into clinical practice. Future research should focus on large, low-bias studies, the identification of standardized cut-off values, the development of adjusted nomograms, and the validation of emerging diagnostic technologies. This systematic review is not registered, as PROSPERO currently does not support registration of diagnostic test accuracy studies.

## Introduction and background

Sepsis- a life-or-death condition- remains a leading cause of morbidity and mortality within the neonatal population [1]. According to the World Health Organization, an estimated 3 million newborns (22 per 1000 live births) are affected [2]. Follow-up studies have shown that survivors of neonatal sepsis can face lasting neurodevelopmental problems such as cerebral palsy, slower mental and physical development, and vision issues [3].

Neonatal sepsis refers to a systemic inflammatory response to a bacterial infection that occurs in infants during the first 28 days after birth. It is divided into two categories: early-onset sepsis (EOS), which occurs within 72 hours of birth, and late-onset sepsis (LOS), which occurs after 72 hours of birth [2,3]. Neonatal sepsis continues to be a diagnostic challenge due to the early signs and symptoms being subtle and non-specific [3]. The vague signs and symptoms can be shared with non-infectious conditions or be a sign of suboptimal transition to postnatal life, such as transient tachypnea of the newborn, respiratory distress syndrome, and apnea of prematurity [3,4].

To assist in diagnosing neonatal sepsis, various laboratory tests are carried out. These are categorized into acute-phase proteins, hematological parameters, cell surface antigens, proteoglycans, cytokines and chemokines, soluble adhesion molecules, and others, e.g., lactate and erythrocyte sedimentation rate (ESR). The gold standard remains the blood culture [4-8]. However, there are some fundamental limitations and challenges. False-negative results can occur due to the following factors: maternal antibiotic use, empirical initiation of antibiotics in neonates before blood samples are collected, small blood volume samples, or low or intermittent bacteremia levels. Conversely, false positives can often occur as a result of contamination during sample collection. Lastly, a significant drawback is the delay in obtaining conclusive results. These results can take 3 to 5 days, thus potentially impacting timely clinical decision-making [4,7,9].

These limitations and challenges, along with the diagnostic difficulties associated with neonatal sepsis, can result in increased antibiotic use. While antibiotics are essential in treating neonatal sepsis, their use also carries inherent risks. The number needed to treat (NNT) to prevent a single confirmed case of EOS in term and late-preterm infants varies from 40 to more than 100, as reported in the literature. The rising concern of antibiotic resistance, driven by overuse, is well-established, with the World Health Organization recognizing it as one of the significant health challenges to address over the next decade [4,8].

Accurate and timely diagnosis of neonatal sepsis is crucial to reduce mortality whilst preventing unnecessary and potentially harmful antibiotic exposure in uninfected neonates [4,6,10]. This is where biomarkers can play a crucial role. An optimal biomarker should exhibit high sensitivity and high specificity, as well as strong positive and negative predictive values [4,5]. It should accurately distinguish septic neonates from those with non-septic illnesses [10].

C-reactive protein (CRP) is the most frequently used laboratory biomarker to assist in detecting neonatal sepsis [2,3,5,6]. CRP is classified as an acute-phase protein, which is primarily synthesized by the liver, but can also be synthesized in the kidney and atherosclerotic tissues. It functions by binding to bacteria, which initiates the attachment of complement proteins and results in phagocytosis [2,3,5,11]. CRP can be influenced by several factors, which can impact its accuracy in detecting neonatal sepsis. These include delayed rise, physiological rise, influenced by gestational age, and influenced by non-septic illnesses [1,3,5,12].

Procalcitonin (PCT) is normally produced by the C cells of the thyroid gland, where it is initially synthesized as pre-procalcitonin, which is then processed by endopeptidases to form PCT. PCT is then converted to calcitonin, a hormone involved in calcium homeostasis. In the presence of bacterial infection, PCT levels can rise 100- to 1000-fold due to the release of endotoxins and cytokines. On the other hand, it can be downregulated by cytokines released secondary to viral infections. PCT can also be produced externally to the thyroid gland, for example, in the liver, pancreas, lungs, kidneys, intestines, and leukocytes. However, production in these tissues is downregulated when no bacterial infection is present. The quicker rise and specificity of bacterial infection are both promising features of this biomarker in aiding the diagnosis of neonatal sepsis [8,10,12-14].

The purpose of this systematic review is to answer, using evidence-based research, the following question: How do PCT and CRP compare in their diagnostic accuracy and clinical utility for the early diagnosis of neonatal sepsis?

## Review

Methods

This systematic review was conducted as per the Preferred Reporting Items for Systematic Reviews and Meta-Analyses (PRISMA) 2020 guidelines [15]. The clinical question of this systematic review utilized the PICO format: P (patient, population, problem) - neonates with sepsis, I (intervention or exposure) -use of C-reactive protein in the detection of neonatal sepsis, C (comparison) - use of procalcitonin in the detection of neonatal sepsis and O (outcome)- early diagnosis (within 48 hours) of neonatal sepsis.

Inclusion and Exclusion Criteria

In this systematic review, papers published between 2020 and 2025 in English, based on neonatal human studies, and with free full text, were reviewed to reveal the most precise results. The included articles contained information regarding neonates who underwent CRP and PCT testing to diagnose neonatal sepsis. Papers published before 2020, in foreign languages, and based on non-human studies were excluded. Additionally, all conference abstracts, posters, and presentations that lacked free full-text articles were excluded because of the limited availability of comprehensive data. Studies with patients who did not undergo CRP or PCT testing to support neonatal sepsis diagnosis were also excluded.

Database and Search Strategy

A literature search was conducted, from May 1 to May 19, 2025, to identify relevant articles comparing the accuracy of C-reactive protein and procalcitonin in diagnosing neonatal sepsis. We utilized the following databases: PubMed/Medline, PubMed Central, Europe PMC, Science Direct, and Google Scholar. Furthermore, to ensure that all relevant articles were identified, we implemented the following strategies: using keywords with the Boolean technique, conducting advanced searches, and using Medical Subject Headings (MeSH) phrases. The keywords used in the search were C-reactive protein, procalcitonin, and neonatal sepsis. Subsequently, we utilized EndNote (Clarivate, London, UK) as a reference manager to facilitate the removal of duplicate articles. The titles and abstracts of the remaining articles were screened, and papers that did not meet our inclusion criteria were removed. In the final stage, the remaining free full-text articles were analyzed against their relevant quality assessment tools. The screening process was conducted independently by two authors, and any disagreements were resolved through discussion until consensus was reached. The detailed search strategies are outlined in Table 1.

**Table 1:** Detailed table displaying search strategies utilized in literature search.

Database	Keywords	Search strategy	Filter	Search result
PubMed	Procalcitonin and C-reactive protein and neonatal sepsis	Procalcitonin AND C-reactive protein AND neonatal sepsis	Last 5 years; free full text; full text; humans; English	27
PubMed MeSH/Medline/PMC	Procalcitonin and C-reactive protein and neonatal sepsis	Procalcitonin OR ( "Procalcitonin/analysis"[Majr] OR "Procalcitonin/blood"[Majr] OR "Procalcitonin/economics"[Majr] OR "Procalcitonin/therapeutic use"[Majr] ) AND C-Reactive Protein OR ( "C-Reactive Protein/analysis"[Majr] OR "C-Reactive Protein/economics"[Majr] OR "C-Reactive Protein/therapeutic use"[Majr] ) AND Early onset neonatal sepsis OR ( "Neonatal Sepsis/blood"[Majr] OR "Neonatal Sepsis/diagnosis"[Majr] OR "Neonatal Sepsis/microbiology"[Majr] OR "Neonatal Sepsis/pathology"[Majr] OR "Neonatal Sepsis/physiopathology"[Majr] )	Last 5 years; free full text; full text; humans; English	235
PubMed Advanced	Procalcitonin and C-reactive protein and neonatal sepsis	((procalcitonin[Title/Abstract]) AND (c-reactive protein[Title/Abstract])) AND (neonatal sepsis[Title/Abstract])	Last 5 years; free full text; full text; humans; English	19
PubMed Central	Procalcitonin and C-reactive protein and neonatal sepsis	-	2020-2025; associated data, MEDLINE journals;	604
PubMed Central Advanced	Procalcitonin and C-reactive protein and neonatal sepsis	((C-reactive protein[MeSH Terms]) AND Procalcitonin[MeSH Terms]) AND neonatal sepsis[MeSH Terms]	Last five years	7
Europe PMC	Procalcitonin and C-reactive protein and neonatal sepsis	(procalcitonin and C-reactive protein and neonatal sepsis AND (HAS_FT:Y OR (HAS_FREE_FULLTEXT:Y))) AND (FIRST_PDATE:[2020-05 TO 2025-05]) AND (((SRC:MED OR SRC:PMC OR SRC:AGR OR SRC:CBA) NOT (PUB_TYPE:"Review")) OR PUB_TYPE:REVIEW)	Free full text; 2020-2025; research articles; review articles	8
Science Direct	Procalcitonin and C-reactive protein and neonatal sepsis	Procalcitonin AND C-reactive protein AND neonatal sepsis	Last 5 years; review articles; research articles	317
Google Scholar	Procalcitonin and C-reactive protein and neonatal sepsis	Procalcitonin AND C-reactive protein AND neonatal sepsis	Last five years; English language; review articles	517

Risk of Bias Assessment

Following screening, the remaining 16 papers were independently reviewed by two individual authors for quality using study-specific quality assessment tools. This includes Assessment of Multiple Systematic Reviews 2 (AMSTAR2) for systematic reviews; Risk of Bias in Non-randomized Studies - of Interventions (ROBINS-I) for non-randomized studies; Scale for the Assessment of Narrative Review Articles (SANRA) for narrative reviews; Quality Assessment of Diagnostic Accuracy Studies 2 (QUADAS-2) for diagnostic accuracy studies, and Newcastle-Ottawa Scale (NOS) for observational studies. Any disagreements were resolved through discussion, and studies scoring 70% or higher were included in the final analysis.

Registering a Systematic Review on PROSPERO

PROSPERO currently does not support the registration of diagnostic test accuracy studies.

Limitations

There are some limitations during this systematic review that should be noted. As part of our exclusion criteria, studies that were older than 2020, not in the English language, and for which free full-text articles were not available were not included. This could have potentially discarded some studies with potentially relevant conclusions. Furthermore, some of the included studies had small sample sizes, different definitions of sepsis, and different cut-off values.

Results

*Study Selection* 

**Figure 1:**
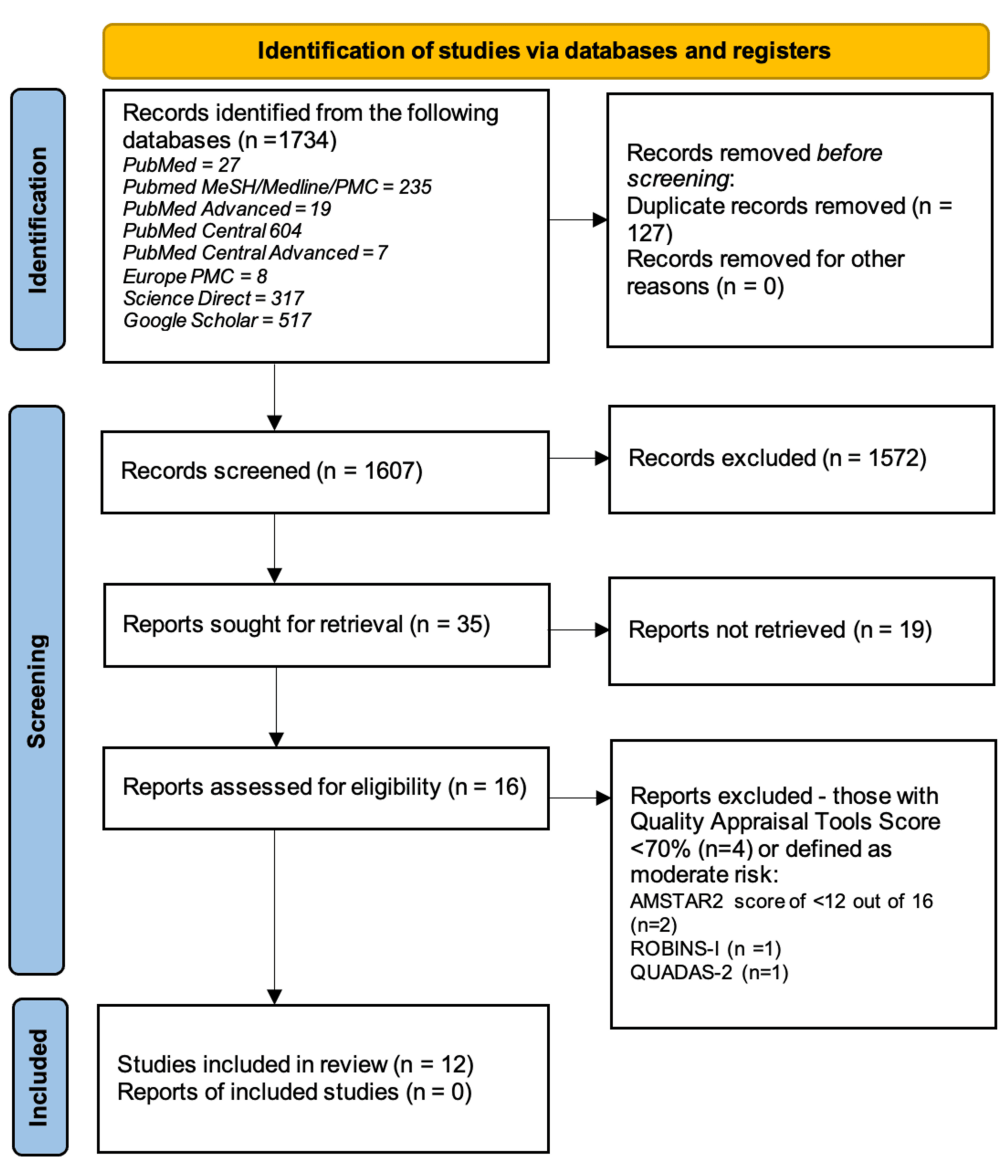
PRISMA flowchart. PRISMA: Preferred Reporting Items for Systematic Reviews and Meta-Analyses; AMSTAR2: Assessment of Multiple Systematic Reviews 2; ROBINS-I: Risk of Bias in Non-randomized Studies - of Interventions; QUADAS-2: Quality Assessment of Diagnostic Accuracy Studies 2.

Using the search strategies detailed in Table 1, a total of 1734 articles were identified from five databases. Following the removal of 127 duplicate records, 1607 records remained. A systematic screening was conducted on the titles and abstracts of the 1607 records to identify those that met the predetermined inclusion criteria, and 35 reports were deemed potentially suitable for further review. The full texts of the 35 reports were reviewed, of which 19 were considered unsuitable, resulting in a final inclusion of 16 studies. Figure 1 illustrates the PRISMA flowchart, detailing the process.

Quality Evaluation

A comprehensive quality assessment was conducted on the remaining 16 free full-text reports using quality assessment tools tailored to each study type. Consequently, 11 studies were included as they were classified as high-quality with a score greater than 70% or a low risk of bias. This detailed process is presented in Table 2.

**Table 2:** Detailed analysis of included studies against relevant quality assessment tools. AMSTAR2: Assessment of Multiple Systematic Reviews 2; ROBINS-I: Risk of Bias in Non-randomized Studies - of Interventions; SANRA: Scale for the Assessment of Narrative Review Articles; QUADAS-2: Quality Assessment of Diagnostic Accuracy Studies 2; NOS: Newcastle-Ottawa Scale.

Quality assessment tool	Type of study	Total score	Accepted score (>70%) or low risk	Accepted study title
AMSTAR2	Systematic reviews	16	12	Procalcitonin for the diagnosis of sepsis in neonates: a diagnostic test accuracy review - Srinivasan et al. (2023) [10].
ROBINS-I	Non-randomized studies	-	Low risk	Nil accepted
SANRA	Narrative reviews	12	9	Noninfectious influencers of early-onset sepsis biomarkers – Tiozzo et al. (2022) [1]. Biomarkers of neonatal sepsis: where we are and where we are going – Boscarino et al. (2023) [3]. Biomarkers of neonatal sepsis: from being mere numbers to becoming guiding diagnostics – Gude et al. (2022) [5]. Early detection of sepsis in neonates: a literature review – Lestari et al. (2023) [6]. The role and validity of diagnostic biomarkers in late-onset neonatal sepsis – Mwesigye et al. (2021) [4]. Advancement in biomarker based effective diagnosis of neonatal sepsis – Gopal et al. (2023) [2]. A comprehensive review of advances in biomarkers for the early diagnosis and management of neonatal sepsis – Mero et al. (2023) [16].
QUADAS-2	Diagnostic accuracy studies	-	Low risk	Diagnostic accuracy of serum procalcitonin (PCT) as an early biomarker of neonatal sepsis using blood culture as gold standard – Habib et al. (2021) [7]. Diagnostic accuracy of point-of-care (POC) testing of C-reactive protein, interleukin-6 (IL-6), and procalcitonin in neonates with clinically suspected sepsis: a prospective observational study – Goyal et al. (2024) [13]. C-reactive protein, procalcitonin, and white blood count to rule out neonatal early-onset sepsis within 36 hours: a secondary analysis of the Neonatal Procalcitonin Intervention Study – Stocker et al. (2021) [8].
NOS	Observational studies	9	7	Risk factors and predictive markers for early and late-onset neonatal bacteremic sepsis in preterm and term infants – Tang et al. (2022) [12].

 Table 3 summarizes the key characteristics of the included studies.

**Table 3:** Study characteristics of included studies. CRP: C-reactive protein; EOS: early-onset sepsis; FBC: full blood count; IL-6: interleukin-6; LOS: late-onset sepsis; PCT: procalcitonin; POC: point-of-care; VLBW: very low birth weight; WBC: white blood cell count.

Study	Study type	Objective	Conclusion/limitations
Srinivasan et al. (2023) [10]	Systematic review	To evaluate the diagnostic accuracy of PCT in culture proven EOS and LOS cases, as well as across all sepsis cases at commonly used cut-off ranges.	Some studies concluded that infants being treated for suspected EOS and randomized to PCT guided algorithm received shorter antibiotics course than standard care. Limitations include unique neonatal physiology, PCT kinetics and requirement for further research into safety utility and economic impact of PCT use in clinical practice.
Tiozzo et al. (2022) [1]	Narrative review	To review the noninfectious conditions and patient characteristics that affect common inflammatory markers used for diagnosis of EOS	Non-infectious factors (perinatal and infant-related) can elevate sepsis biomarkers, and their use without full clinical or microbiological confirmation risks antibiotic overuse and associated harms.
Boscarino et al. (2023) [3]	Narrative review	To review findings and assess the early diagnostic accuracy of currently used biomarkers for neonatal sepsis.	Combination of 2 or more current biomarkers has been suggested as more productive in aiding diagnosis and thus use of antibiotic therapy. Limitations include difficulty to determine cut-off levels due to non-ideal kinetics (particularly CRP), as well as being influenced by pre-, peri-, and postnatal factors.
Gude et al. (2022) [5]	Narrative review	To review the function, advances, and outlook of various biomarkers in early diagnosis, treatment of and prognosis of neonatal sepsis.	No single ideal biomarker was found. CRP described as a specific for neonatal sepsis with normal levels ruling it out and sensitivity increasing with serial values. Benefit of PCT in the diagnosis of EOS is its earlier rise and rise being independent of gestational age. Limitations of CRP in diagnosing EOS include its slow rise (late biomarker), long half-life and rise in non-infectious conditions. PCT limitations include rise in non-infectious conditions (like CRP) and inability for sole use to diagnose neonatal sepsis. Improvements include the use of a lower cut-off value.
Lestari et al. (2023) [6]	Narrative review	To identify and evaluate, using evidence-based methods, the most effective and optimal methods for early diagnosis of neonatal sepsis.	PCT, compared to CRP, had increased diagnostic accuracy in EOS. Both CRP and PCT rose in preterm infants with sepsis. Combination of CRP and FBC can be used to diagnose LOS, especially in VLBW infants. Use of PCT has been promising in developing countries with low socioeconomic conditions.
Mwesigye et al. (2021) [4]	Narrative review	To review and highlight the current work on biomarkers and their role specifically in LOS.	Sensitivity of CRP, with regards to LOS diagnosis, increases with combined with FBC. Although PCT has a natural postnatal rise, it rises rapidly post-infection and remains elevated for up to 48h. Its level can indicate severity of illness and reduce rapidly post antibiotic use. PCT is undetectable in healthy individuals, thus demonstrating its specificity.
Gopal et al. (2023) [2]	Narrative review	To evaluate recent biomarkers, thus identifying a promising biomarker and its potential use in supporting the development of electrochemical biosensors for POC neonatal sepsis detection.	Diagnostic accuracy of CRP increases when used in combination with other biomarkers. Limitations include short half-life, low specificity and sensitivity and false positives due to rise in non-infectious conditions. PCT rises rapidly in infection and stays elevated for next 24. Its level represents severity of infection. Despite its limitations, which include high cost and expression affected by various factors, it is considered superior.
Mero et al. (2023) [16]	Narrative review	To discuss recent progress and utilization of biomarkers in early detection and management of neonatal sepsis.	Limitation of CRP, which reduce its sensitivity, include slow rise and rise in non-infectious conditions. Early rise of PCT makes it useful in EOS diagnosis. PCT limitations include that its rise is reportedly affected by gestational age. Improvements in PCT use include having age-specific nomograms and use in combination with other biomarkers.
Habib et al. (2021) [7]	Diagnostic accuracy study	To analyze the diagnostic accuracy of PCT as a biomarker for early detection of neonatal sepsis prior to confirmation by blood culture.	Early availability of PCT result (2h) demonstrated it to be a good aid in early and prompt diagnosis and treatment of neonatal sepsis. Study limitations include absence of serial PCT values, prognostic value of various levels of PCT at initial presentation, knowing causative organism in neonatal sepsis and small sample size.
Goyal et al. (2024) [13]	Diagnostic accuracy study	To compare the diagnostic accuracy and correlation between POC and laboratory CRP for sepsis, and to review the accuracy of POC CRP, IL-6, and PCT for EOS and LOS.	Good correlation between laboratory and POC CRP measurements. CRP shows optimum diagnostic accuracy both when solely utilized but also when with PCT. The diagnostic accuracy of POC remains at a similar level in both EOS and LOS. The POC test for IL-6, CRP and PCT can be carried out in ~12 mins, drastically quicker than standard laboratory techniques.
Stocker et al. (2021) [8]	Diagnostic accuracy study	To evaluate serial CRP, PCT and WBC measurements, focusing on negative predictive values, to identify the safest time to discontinue antibiotics in suspected EOS, and aid future research on reducing unnecessary antibiotic exposure.	CRP performed better but need to consider its inclusion in the definition of uncertain and probable sepsis cases. CRP had a physiological rise. PCT limitations include its cost and kinetics (which require nomogram use). Normal CRP and PCT, within 36h starting empirical antibiotic initiation confidently support their discontinuation. Benefit seen in combining PCT and CRP. Difficulty of culture-negative sepsis management discussed. Study limitations include its design as a secondary analysis (potential bias introduction) and small sample size of proven sepsis cases.
Tang et al. (2022) [12]	Observational study	To evaluate bacteremia risk factors and identify predictive marker cut-off for diagnosis of EOS and LOS in both term and preterm infants.	PCT limitations include rise in non-bacterial causes, debated cut-off values and inability to differentiate in LOS cases irrespective of bacteremia, thus need further research into serial measurements for LOS. Elevated CRP is a risk factor of bacteremia in EOS but not LOS (no significance between bacteremia and non-bacteremia). Serial CRP measurements suggested due to delayed serum production. CRP limitations include susceptibility to gestational age and difficulty distinguishing physiological rise from bacterial infection. Study limitations include small sample size and study design.

Discussion

The identification of an ideal sepsis biomarker remains a key clinical priority. This systematic review demonstrates the strong diagnostic potential of PCT, particularly in the early diagnosis of neonatal sepsis. Furthermore, the diagnostic accuracy of PCT is enhanced when used in combination with other biomarkers, including CRP. These findings carry significant clinical implications. They support the incorporation of PCT into current sepsis detection methods, reduce unnecessary antibiotic exposure (antibiotic stewardship), and ultimately support accurate, evidence-based neonatal care.

Early-Onset vs. Late-Onset Sepsis

Neonatal sepsis can be subdivided into early-onset and late-onset sepsis. Early-onset sepsis occurs within 72 hours of birth, compared to late-onset sepsis, which occurs more than 72 hours after birth. Table 4 summarizes the timing, causative pathogens, and associations that differentiate early-onset sepsis (EOS) and late-onset sepsis (LOS) [2-6,10,12,16-18].

**Table 4:** Summary of EOS and LOS characteristics focusing on timing, causative pathogens, and their mode of transmission. EOS: early-onset sepsis; LOS: late-onset sepsis.

Feature	EOS	LOS
Timing	<72h after birth	>72h after birth
Causative pathogens	Group B streptococcus, Escherichia coli, Streptococcus viridans, Enterococci, Staphylococcus aureus, Pseudomonas aeruginosa, Listeria monocytogenes, Haemophilus influenza, coagulase negative staphylococcus.	Bacterial: Coagulase-negative staphylococci, Group B streptococcus, Staphylococcus aureus, Acinetobacter baumanii, Candida albicans, Klebsiella pneumonia, Escherichia Coli, Enterococci, Pseudomonas aeruginosa; Viral: echovirus, enterovirus, parechovirus, coxsackie, adenovirus, parainfluenza, rhinovirus, coronavirus; Fungal: Candida.
Mode of transmission	Vertical pathogen transmission from maternal genitourinary tract to fetus: placental transmission, ascending infection from vagina to uterus from rupture of amniotic membrane during delivery.	Postnatal nosocomial infections: invasive procedures which disturb mucosa, intravascular devices. e.g., central venous catheters contact from healthcare workers or caregivers. Some may be due to late presentation of vertically transmitted infection.

**Figure 2:**
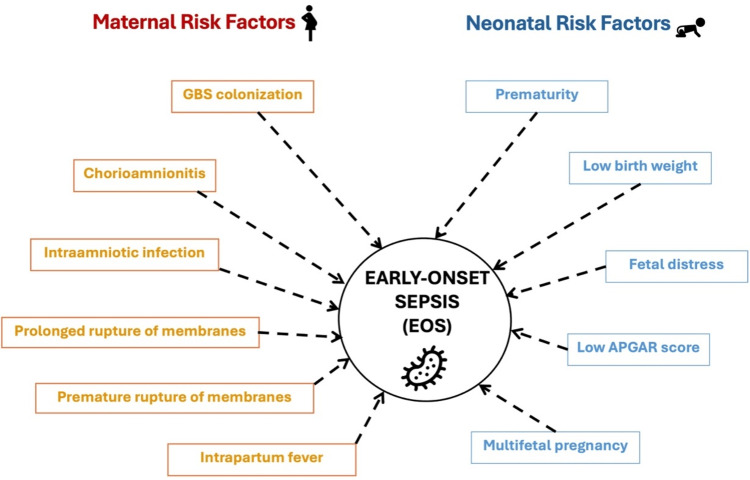
Risk factors of early-onset sepsis. GBS: group B streptococcus.

**Figure 3:**
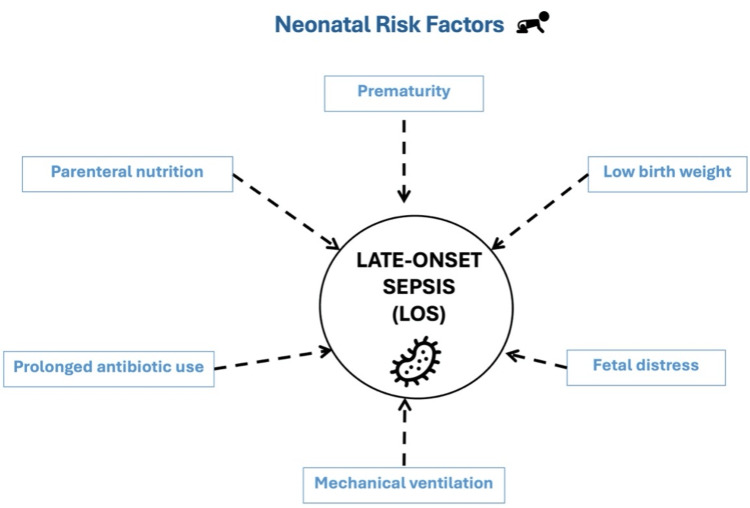
Risk factors of late-onset sepsis.

Several risk factors are associated with EOS and LOS. These can be categorized into maternal and neonatal risk factors. Figure 2 and Figure 3 illustrate these points, respectively [2-6,12,16-18].

Biomarkers in Current Clinical Use

Numerous biomarkers have been utilized to aid in the diagnosis of neonatal sepsis. These biomarkers can be categorized into the following: acute-phase proteins, hematological parameters, proteoglycans, cytokines and chemokines, soluble adhesion molecules, and others. Table 5 summarizes the biomarkers currently utilized, as reported in the included studies.

**Table 5:** Biomarkers in current clinical practice. CRP: C-reactive protein; PCT: procalcitonin; SAA: serum amyloid protein; WBC: white blood cell; ANC: absolute neutrophil count; I/T ratio: immature to total leukocyte ratio; CD: cluster of differentiation; IL: interleukin; TNF-α: tumour necrosis factor-alpha; sTNF-R: soluble tumour necrosis factor receptor; ICAM: intercellular adhesion molecule; ESR: erythrocyte sedimentation rate.

Biomarker category	Biomarker
Acute phase proteins	CRP
	PCT
	SAA
	Fibrinogen
	Ferritin
	Complement
Hematological parameters	WBC
	Total leukocyte count
	ANC
	I/T ratio
	Platelet count
Cell surface antigens	CD64
	CD11b
	Presepsin
Proteoglycan	Endocan
Cytokines & chemokines	IL-1
	IL-6
	IL-8
	IL-10
	TNF-α
	sTNF-R
Soluble adhesion molecules	ICAM
Other	Lactate
	ESR

Accuracy of C-reactive Protein in Diagnosing Neonatal Sepsis

Limitations of CRP: CRP is a widely used biomarker in the detection of neonatal sepsis; however, some limitations reduce its diagnostic accuracy in the early detection of neonatal sepsis [2,3,5,6]. 

CRP exhibits a delayed rise, typically 6-12 hours after the onset of infection, and a short half-life of 24-48 hours [1-3,5,12,16]. This limitation lowers its sensitivity, particularly for the diagnosis of EOS, often resulting in prolonged empirical antibiotic use and associated risks [1,2,12,16]. 

Furthermore, the kinetics of CRP can be affected by various perinatal and infant risk factors. Perinatal risk factors include vaginal delivery, duration of labour, rupture of membranes, maternal fever or signs of infection, maternal intrapartum antibiotic prophylaxis, and maternal hypertensive disorder. 

Infant risk factors include lower gestational age, increased birth weight, birth injury, e.g., cephalohematoma and tissue trauma, hypoxic-ischemic encephalopathy, meconium aspiration syndrome, gastroschisis, surgery, preterm exposure to steroids, surfactant administration, and pneumothorax [1,3,5,16]. Premature infants had lower CRP levels, with a rise of 0.405 mg/l for every one-week increase in gestational age, and a slight rise was noted during infection. There is also a physiological rise during the first 72 hours after birth. Several of the included studies have concluded that the above factors increase the difficulty of establishing a single cut-off value [3,19].

Advantages of CRP: Despite its known limitations, CRP remains a valuable tool in the diagnosis of neonatal sepsis due to its simplicity, rapid turnaround time, low cost, and widespread availability [19]. 

CRP values can be monitored, and normal CRP values indicate the absence of bacterial infection, allowing clinicians to safely discontinue antibiotics [3,5,19]. CRP had low sensitivity but higher specificity [4]. Compared to a singular value, serial CRP values (within 24-48 hours of disease onset) have been found to increase sensitivity in neonatal detection and subsequent assessment of response to treatment [1,3,5,13,19]. An increase in sensitivity, from 35% at onset to 44% and 54%, was noted in the detection of culture-positive EOS when samples were taken at 8-24 hours and 8-48 hours after onset [3].

Moreover, the included studies emphasized the benefit of utilizing CRP in conjunction with other sepsis biomarkers to increase the accuracy of EOS and LOS diagnosis [2,3,5,13]. In a study by Beltempo et al., the authors noted an increase in sensitivity when CRP was combined with a complete blood count (CBC) for the detection of LOS [4]. Individually, CRP had a higher sensitivity value of 89%, while CBC had a sensitivity of 59%. However, when used together, the combined sensitivity was 88% with a negative predictive value of 93% [4,6]. Other potential biomarkers that can be used in conjunction with CRP include CD64, interleukins, or PCT [13,19]*. *

Improvements in CRP use: Suggestions to improve CRP use in the detection of neonatal sepsis include recognizing non-infectious conditions that affect CRP levels, and being aware of these conditions during the interpretation of values. Furthermore, testing should be omitted in cases where there is a raised likelihood of sterile inflammation, where the use of a biomarker would not differentiate additional infection. Lastly, the use of adjusted nomograms with different cut-off values for gestational or chronological age, as well as mode of delivery [1,3,19]. 

This systematic review suggested the following future directions for the use of CRP in diagnosing neonatal sepsis. These include the use of POC testing, salivary CRP, and highly sensitive-CRP (hs-CRP) [2,5,13]. It was found that POC CRP matched closely with laboratory values, and the mean turnaround time is almost 12 minutes, which is much quicker than traditional techniques [13]. Some studies have proposed the use of salivary CRP as an alternative; however, further research is required to enable its large-scale use [2]. Lastly, hs-CRP has higher sensitivity than traditional CRP and a lower cut-off value, therefore displaying increased sensitivity for suspected neonatal sepsis [5].

Accuracy of Procalcitonin in Diagnosing Neonatal Sepsis

Advantages of PCT*: *PCT has emerged as a promising biomarker in the early detection of neonatal sepsis. It is important to note that PCT exhibits a physiological rise after birth, reaching peak values around 24 hours postnatally before declining by 48-72 hours of age. This is dependent on gestational age, with an inverse relationship noted between gestational age and height of PCT response [3]. Another study stated that the rise occurs two to four days after birth [4]. However, in comparison to CRP, PCT is released into the bloodstream very quickly after systemic bacterial exposure (both EOS and LOS), within two to four hours, reaches its peak within six to eight hours, and remains raised for up to 48 hours [2-5,7,12,13]. The half-life of PCT is 24-30 hours [5,16]. This early rapid rise makes PCT a valuable marker for the early diagnosis of neonatal sepsis [5,16]. Diagnostic accuracy increased when serial values were monitored [3].

Some studies highlighted its benefit, particularly in LOS, with higher diagnostic accuracy [3,5,19]. One study concluded that at this stage, the physiological changes stated above no longer affect levels, and thus can accurately inform of bacterial infection, with sensitivity and specificity values of greater than 80% [3]. Another study exploring LOS stated a sensitivity of 88%, specificity of 71.4%, and a negative predictive value of 87% in cases of confirmed sepsis [5]. Conversely, another study concluded that PCT values did not differ significantly between bacteremia and non-bacteremia groups in LOS [12].

PCT is described as a good and specific marker for differentiating bacterial or fungal infections from viral infections, as it doesn’t demonstrate an appreciable increase in viral infection [12,13,20]. It has been suggested that this is due to the release of interferon-γ in viral infection, which suppresses PCT release [3]. A study focusing on the use of PCT in LOS highlighted the benefit of using PCT in differentiating bacterial infections from fungal infections, as it found no significant difference in PCT values in the diagnosis of fungal infections [21].

PCT is essentially undetectable in healthy individuals (0.033- 0.046 ng/ml), and if consistently low, it rules out EOS and LOS [3,4,7]. It was also seen to rule out blood culture contamination from true infections [20].

Regarding its sensitivity and specificity, PCT demonstrated higher sensitivity but lower specificity than CRP [4,15,22]. Habib et al. stated a sensitivity of 97.7% compared to a specificity of 70.6%, a negative predictive value (NPV) of 96.8%, and a positive predictive value (PPV) of 77.1% [7]. This was echoed by Morad et al., where PCT had a sensitivity of 97.6%, a specificity of 89%, a PPV of 97.6%, and an NPV of 88.9% [20].

This review found that PCT was beneficial in guiding antibiotic therapy. Srinivasan et al. noted that neonates being treated for suspected EOS and allocated to PCT-guided therapy received a shorter duration of antibiotic therapy compared to standard care [10].

PCT values also indicate severity, and they quickly decrease after the start of antibiotic therapy. Once values return to normal levels, antibiotics can be stopped [2-4,7,23]. A secondary analysis of the Neonatal Procalcitonin Intervention Study concluded that normal CRP and PCT within 36 hours after starting antibiotic therapy confidently excludes EOS, as there is no increase in NPV of both from 36 hours to 48 hours [8]. Included studies suggested the use of PCT in conjunction with other biomarkers [3,5,16,20,21].

Limitations of PCT*: *Difficulty arises in determining the cut-off value for optimum diagnostic accuracy. PCT can be affected by non-infectious conditions stated previously under "Accuracy of C-reactive Protein in Diagnosing Neonatal Sepsis" [1,3,5]. Perinatal factors had less effect on PCT values compared to infant characteristics [1].

PCT can exhibit different behavior depending on the specific pathogen in question. Thus, its diagnostic accuracy can also be affected, both directly and indirectly, not only by clinical symptoms but also by the local unit's microbiological profile [5].

Furthermore, one study reported that PCT is not affected by gestational age in bacterial sepsis [5]. Conversely, a study disputed this and found that values in neonates <32 weeks’ gestational age can be changed, so they need to be interpreted with care and suggested gestational age-specific nomograms [16].

It has been suggested that having lower cut-off levels for neonatal sepsis diagnosis could increase its sensitivity and specificity [5]. Having a cut-off value ≥0.5 ng/ml displayed the highest sensitivity, specificity, PPV, and NPV [7,20].

PCT’s high cost has been noted in several studies [2,8,21]. However, it was found that the difference, compared to standard care, was not significant [23].

Comparing the Diagnostic Performance of CRP and PCT at Various Cut-Offs

CRP demonstrates moderate sensitivity (75-100%) and specificity (49.18-86.7%) across the different cut-off values. The highest sensitivity (100%) was observed at 16 mg/L, and the highest specificity (86.7%) was achieved at 6.4 mg/L. The negative predictive value (NPV) and positive predictive value (PPV) vary, and there is insufficient data to compare them at various cut-offs.

PCT shows higher sensitivity overall (100% at 2.8 ng/L) and good specificity, ranging from 70.6% to 95%. A cut-off limit of >0.5ng/ml demonstrated a two-fold probability of sepsis [24]. A cut-off value of > 2 ng/mL shows an optimum diagnostic balance with a sensitivity of 87.2%, specificity of 72.13%, and a high NPV (89.8%).

The findings within the study, published in the American Academy of Pediatrics, align with those of this systematic review [22]. They report that PCT had a high sensitivity (87-100%) when a cut-off value of 0.5 ng/mL was used; however, specificity was more variable across the cut-off values analyzed [22].

Table 6 summarizes the normal and neonatal sepsis-associated values for both PCT and CRP, along with their sensitivity, specificity, PPV, and NPV at various cut-off values.

**Table 6:** Table summarizing the diagnostic performance of PCT and CRP. PCT: procalcitonin; CRP: C-reactive protein; PPV: positive predictive value; NPV: negative predictive value.

Biomarker	Normal level	Level in neonatal sepsis	Cut-off value	Sensitivity (%)	Specificity (%)	PPV (%)	NPV (%)	Reference
CRP	0-3mg/l [2]	12-48mg/l [2]	3mg/l	82.5	77.5	88	68.9	[25]
			6.4mg/l	90	86.7	-	-	[6]
			10mg/l	76.92	49.18	49.18	76.92	[26]
			15mg/l	75	84	14	99	[12]
			16mg/l	100	-	-	-	[8]
PCT	<0.5ng/ml [19]	>2-2.5ng/ml [19]	0.5ng/ml	97.7	70.6	-	-	[7]
			1ng/ml	86	75.3	-	-	[7]
			1.5ng/ml	77.9	77.6	-	-	[7]
			2ng/ml	72.1	78.8	-	-	[7]
			2.5ng/ml	66.3	81.2	-	-	[7]
			2.8ng/l	100	-	-	-	[8]
			3ng/ml	62.8	82.4	-	-	[7]
			>2ng/dl	87.2	72.13	66.67	89.8	[26]
			27 µg/l	75	95	33	99	[12]

Clinical Decision Flowchart

**Figure 4:**
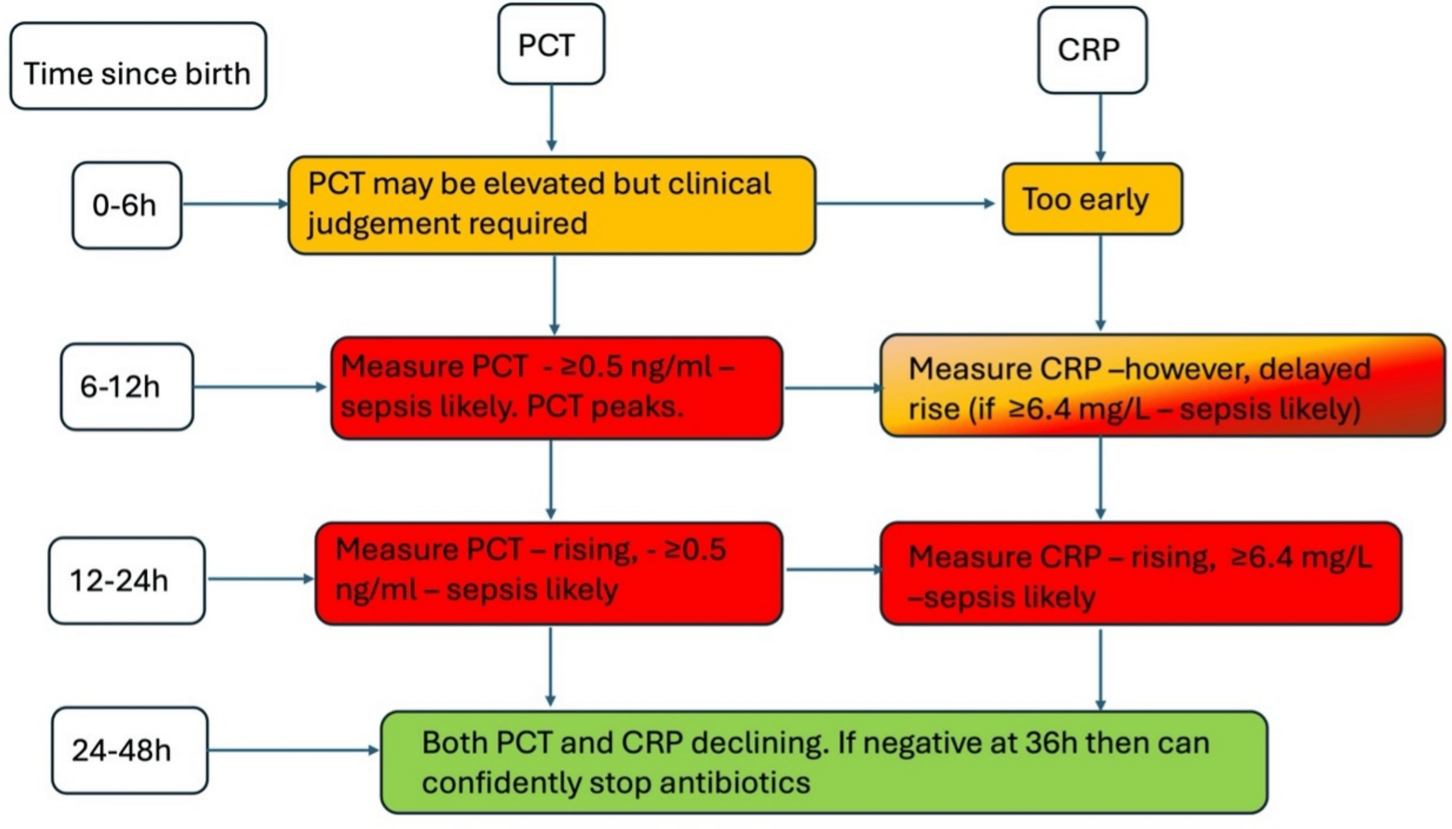
Clinical decision flowchart demonstrating recommended optimum timing and cut-off values. PCT: procalcitonin; CRP: C-reactive protein.

During our review of major international guidelines (World Health Organization or WHO, Centers for Disease Control and Prevention or CDC, and National Institute for Health and Care Excellence or NICE), we found that while CRP is mentioned in some recommendations (WHO and NICE), PCT is not currently incorporated into any formal pathways to aid the diagnosis of neonatal sepsis. We have also not found a unified guideline in the CDC guideline library [27-29]. This absence highlights both a gap in current guidance and the need for further research to support future recommendations. To address this, and based on the evidence analyzed in this systematic review, we developed a clinical decision-making flowchart (Figure 4), which illustrates a potential application of PCT alongside CRP in the diagnostic evaluation of neonatal sepsis. This proposed diagnostic flowchart is intended to complement existing clinical assessments and could provide a framework for future protocol development once further validation is achieved.

## Conclusions

Neonatal sepsis remains a major cause of morbidity and mortality. Diagnostic challenges have led to increased empirical antibiotic use and its associated risks, including the development of antibiotic resistance. Therefore, identifying biomarkers that can help early and accurate diagnosis of neonatal sepsis is crucial for improving clinical outcomes and optimizing antibiotic stewardship. CRP remains widely used due to its simplicity, speed, and low cost. However, its delayed rise, short half-life, and susceptibility to non-infectious influences limit its effectiveness for early and accurate diagnosis of neonatal sepsis.

On the other hand, PCT rises earlier and remains elevated for 48 hours. They also correlate well with disease severity, with low or undetectable levels effectively ruling out sepsis. It also demonstrated higher sensitivity and can differentiate between bacterial infections and those caused by viruses and fungi. However, its limitations include lower specificity, higher cost, a physiological rise after birth, and susceptibility to non-infectious conditions. Combining CRP and PCT (and with other biomarkers) enhances their diagnostic accuracy. Our review identified that, unlike CRP, procalcitonin is absent from current international protocols for the diagnosis of neonatal sepsis. The proposed flowchart provides a potential framework for integrating PCT into clinical assessment, pending further validation. Future research directions should focus on well-designed studies with a reduced risk of bias and a larger sample size, identifying standardized cut-off values and developing adjusted nomograms that account for various influential factors, as well as the validation of emerging technologies.
